# Preclinical pharmacokinetic modeling and clinical translation of SIG001, a novel anti-cancer therapeutic antibody targeting on cancer-derived sialylated IgG

**DOI:** 10.3389/fphar.2026.1904283

**Published:** 2026-09-01

**Authors:** Qingyu Yao, Chen Liu, Shenghua Zhang, Yaou Liu, Ying Zhou, Xiaoyan Qiu

**Affiliations:** 1 Clinical Trial Center, Beijing Key Laboratory of Clinical Pharmacology and Translation of Innovative Drugs, Peking University First Hospital, Beijing, China; 2 Department of Immunology, School of Basic Medical Sciences, NHC Key Laboratory of Medical Immunology, Medicine Innovation Center for Fundamental Research on Major Immunology-related Diseases, Peking University, Beijing, China; 3 Department of Pharmacy, Peking University First Hospital, Beijing, China

**Keywords:** first-in-human dose, modeling and simulation, pharmacokinetic, sialylated IgG, therapeutic antibody

## Abstract

**Introduction:**

Sialylated immunoglobulin G (SIA-IgG) has emerged as a novel therapeutic target selectively overexpressed in epithelial-derived malignancies. The humanized monoclonal antibody SIG001 binds specifically to tumor-associated SIA-IgG and is regarded as a promising first-in-class candidate for the broad-spectrum treatment of epithelial cancers.

**Methods:**

The pharmacokinetic (PK) behavior of SIG001 was evaluated in both cynomolgus monkeys and mice following intravenous administration. Utilizing modeling approaches, we projected the first-in-human (FIH) starting dose and the human pharmacologically active dose (PAD) from preclinical datasets.

**Results:**

In cynomolgus monkeys, single-dose PK was assessed at 5, 15, and 30 mg/kg, while in mice, doses of 5, 10, and 20 mg/kg were investigated. Data from both species were well characterized by a two-compartment model with linear elimination kinetics; human PK parameters were extrapolated from the monkey studies. *In vitro* studies demonstrated that SIG001, at concentrations up to 50 μg/mL, did not elicit significant cytokine release from human peripheral blood mononuclear cells (PBMCs), establishing this concentration as a safety threshold for FIH dose estimation. Based on simulations incorporating moderate PK variability, a maximum recommended starting dose of 0.15 mg/kg, administered biweekly, was identified. Efficacy studies in murine xenograft models revealed a plateaued, dose-dependent antitumor response across the range of 2.5–10 mg/kg, indicative of maximal receptor occupancy and a saturated therapeutic effect. The translational scaling of murine PK profiles yielded human equivalent doses (HEDs), with predicted PAD values ranging from 1.8 to 7.0 mg/kg.

**Conclusion:**

The preclinical investigations delineate the PK profile and antitumor activity of SIG001. The quantitative translation of animal data into human PK and exposure–response expectations provides a rational foundation for clinical development and supports informed decision-making in the design of early-phase trials.

## 1 Introduction

Immunoglobulins (Igs) are widely recognized as antibody molecules secreted by B-lineage lymphocytes and play an essential role in adaptive immune responses (Honjo and Habu, 1985). In recent decades, however, accumulating evidence has demonstrated that immunoglobulins can also be expressed independently of B cells, including epithelial cells, cardiomyocytes, myeloid cells, and neurons (Zhang et al., 2010; Wang et al., 2015; Zhu et al., 2017; Niu et al., 2011). Moreover, elevated IgG expression has been found in various epithelial malignancies such as lung, breast, colon, esophagus, ovarian, prostate, and bladder cancers (Hu et al., 2025; Liu et al., 2015; Zhang et al., 2012; Niu et al., 2012; Lee et al., 2012; Sheng et al., 2016), and the cancer-derived IgG, namely, SIA-IgG, is characterized by a unique highly sialylated N-glycosylation at asparagine position 162 (Asn162) in the first domain of the heavy chain constant region (CH1) (Tang et al., 2018). SIA-IgG has been shown to induce proliferation and migration by interacting with the integrin α6β4 complex and activating the FAK and Src pathways and to activate c-Met-SOX2 signaling to promote stemness in cancer cells in a self-propagating manner (Tang et al., 2018; Huang et al., 2023). Furthermore, it has also been reported that SIA-IgG plays an important role in tumor immune escape by inhibiting the function of effector T cells (Wang et al., 2020; Fan et al., 2023). Given its widespread expression in malignancies and its critical functions in promoting tumorigenesis, progression, and immune evasion, SIA-IgG emerges as a promising broad-spectrum therapeutic target for epithelial-derived cancers.

SIG001 is a humanized monoclonal antibody that has been modified based on the variable region sequence of the murine anti-SIA-IgG monoclonal antibody RP215, which has been proven to show significant inhibition of tumor growth and enhancement of anti-tumor immune function by specifically binding to the cancer-derived SIA-IgG in preclinical studies (Tang et al., 2018; Huang et al., 2023; Fan et al., 2023). SIG001 is a recombinant protein generated through the humanization of RP215. With a high affinity for SIA-IgG (K_D_ = 6.22 × 10^−9^ M), SIG001 is expected to become the first oncological therapeutic antibody targeting cancer-derived SIA-IgG and is about to enter the early clinical research stage. Thus, it is critical to provide rational dose selection for the first-in-human (FIH) trial design based on non-clinical data.

The aim of this study is to explore the pharmacokinetic (PK) profiles of SIG001 in preclinical species, including cynomolgus monkeys and mice, along with its antitumor efficacy in tumor-bearing mice at multiple dose levels. Human PK properties are predicted based on population modeling approaches with allometric scaling, and simulations are performed to provide helpful insights into the dose design of the FIH trial.

## 2 Methods

### 2.1 Chemicals and reagents

SIG001 was synthesized by Zencore Biologics (Shanghai, China) under a contractual agreement. Intravenous immunoglobulin (IVIG) was acquired from Grand Shuyang Life Science (Chengdu, China). Dulbecco’s modified Eagle medium (DMEM) was sourced from Gibco (CA, United States), fetal bovine serum (FBS) from PAN-Biotech (Bavaria, Germany), and penicillin–streptomycin (100×) from HyClone (DE, United States). Matrigel was obtained from Corning (NY, United States), while phosphate-buffered saline (PBS) was procured from Solarbio (Beijing, China).

### 2.2 Cell culture

The human epidermoid carcinoma cell line A-431, the triple-negative breast cancer cell line MDA-MB-231, and the colorectal cancer cell line SW480 were acquired from the American Type Culture Collection (ATCC). The human pancreatic cancer cell line T3M-4 was generously provided by Dr. Jörg Kleeff (Martin-Luther-University Halle-Wittenberg, Germany). All cell lines were cultured in DMEM supplemented with 10% FBS and 1% penicillin–streptomycin at 37 °C in a humidified incubator with 5% CO_2_.

### 2.3 Animals

Cynomolgus monkeys and CD-1 mice utilized in pharmacokinetic (PK) studies were acquired and housed at WuXi AppTec (Suzhou, China). For pharmacodynamic (PD) studies, athymic nude (nu/nu) and NOD/SCID mice (6–8 weeks old) were obtained from Charles River Laboratories (Beijing, China) and maintained at the Department of Laboratory Animal Science, Peking University Health Science Center. Mice were housed in individually ventilated cages under specific pathogen-free conditions at a temperature of 22 °C–24 °C, with 50%–60% relative humidity, and on a 12-h light/12-h dark cycle. All animal procedures were reviewed and approved by the Institutional Animal Care and Use Committee of Peking University Health Science Center.

### 2.4 Preclinical PK analysis

The concentration data of SIG001 were obtained from cynomolgus monkeys after a single i. v. dose of 5, 15, and 30 mg/kg of SIG001 and 5, 10, and 20 mg/kg for CD-1 mice. Each dose group of cynomolgus monkeys consisted of 3 male and 3 female animals, and blood samples were collected from 6 animals before dosing and at 0.5 h, 1 h, 2 h, 4 h, 8 h, 24 h, 2 days, 3 days, 4 days, 7 days, 10 days, 14 days, 21 days, and 28 days after dosing, and the concentrations were analyzed using an enzyme-linked immunosorbent assay (ELISA) with a lower limit of quantification (LLOQ) of 100 ng/mL. Meanwhile, a crossover sampling strategy was employed in mice due to the limited blood volume, wherein each animal provided blood samples at only two time points, aligning the PK sampling time points with those in the cynomolgus monkeys’ PK study. The concentration data at each time point for each dose group were contributed by 6 different animals, except for the 28 d time point, which included data from 12 animals. At each sampling time, equal numbers of male and female animals were represented, and the concentrations were also analyzed using ELISA with an LLOQ of 100 ng/mL. Non-compartment analysis was conducted using the ncappc package in R (version 4.4.2).

### 2.5 PD studies

*In vivo* studies were carried out to investigate the anti-tumor efficacy of SIG001 in various xenograft models, including A-431, MDA-MB-231, T3M-4, and SW480 cell-derived xenografts (CDXs), along with lung squamous cell carcinoma (LSCC) and pancreatic ductal adenocarcinoma (PDAC) patient-derived xenografts (PDXs). Details on implantation, grouping, dosing, and tumor-volume evaluation are listed in Supplementary Table S1 in Supplementary Material. In brief, mice were implanted with tumor cells or tissues and then randomized into groups to receive either PBS solution (control group) or 2.5, 5, and 10 mg/kg SIG001 via i.v. administrations. Tumor length and width were measured, and tumor volume was calculated: tumor volume = length × width × 0.5236 (Zhang et al., 2010). Animals were sacrificed before the tumor size in the control group reached the threshold permitted by animal ethics guidelines.

### 2.6 Human peripheral blood mononuclear cell cytokine release assay

Human peripheral blood mononuclear cells (PBMCs) from six healthy donors were incubated with PBS, IVIG, RP215, and SIG001 at 50 μg/mL for 96 h, and cytokines, including IL-2, IL-4, CXCL10, IL-1β, TNF-α, CCL2, IL-17A, IL-6, IL-10, IFN-γ, IL-12p70, IL-8, and TGF-β1, were assessed using cytometric bead array (CBA) at 24 h, 48 h, 72 h, and 96 h. Data from the CBA were fitted using the BioLegend website (https://legendplex.qognit.com/) to quantify the fluorescence intensity and the concentration of the cytokines.

### 2.7 Statistics

Tumor growth curves were analyzed using a mixed-effects model (restricted maximum likelihood, REML). Time and treatment group were included as fixed effects, while individual animals were treated as random effects. The interaction between time and treatment was evaluated to determine whether treatment altered tumor growth trajectories. Tukey’s multiple comparisons test was performed for pairwise comparisons among treatment groups. A two-sided *p*-value <0.05 was considered statistically significant.

### 2.8 Development and evaluation of population PK model

The PK profiles of SIG001 in both cynomolgus monkeys and mice were described by a two-compartment model. The modeling and simulation were performed using NONMEM software (version 7.5.0, ICON Development Solutions, Dublin, Ireland) and PsN (version 5.2.6, Uppsala University, Uppsala, Sweden). The first-order conditional estimation with interaction (FOCE-I) method was used in estimation, and M3 was employed in the PK model of mice due to the proportion of concentrations below the quantification limit (BQL) reaching 20% (Ahn et al., 2008). In addition, inter-individual variability (IIV) was incorporated using an exponential error model, while residual variability (RV) was examined through additive, proportional, and combined error structures.

Model selection was based on the physiological plausibility of parameter estimates, the objective function value (OFV), and diagnostic plots. The precision of parameter estimates was evaluated using the relative standard error (RSE). Furthermore, parameter uncertainty was assessed through a bootstrap approach involving 1,000 runs. Additionally, a visual predictive check (VPC) was performed based on 1,000 simulations to evaluate the model’s predictive performance.

### 2.9 Allometric scaling of PK parameters in humans

The PK parameters of SIG001 in humans were predicted based on the parameter estimates from the PK model of cynomolgus monkeys with allometric scaling, including the clearance (CL), the clearance between compartments (Q), the distribution volume of the central compartment (V_C_), and the peripheral compartment (V_P_), as shown in Equation 1:

$$P_{human}=P_{monkey}\times {\left(\frac{{BW}_{human}}{{BW}_{monkey}}\right)}^{\alpha },$$ (1)

where P_human_ and P_monkey_ represent the PK parameters in human and cynomolgus monkey, respectively. BW_human_ stands for the body weight of human, which is assumed to be 70 kg. BW_monkey_ was assigned a value of 2.24 kg, which was the mean body weight of the cynomolgus monkey employed in the PK study. In addition, α is the scaling factor, assigned a value of 0.8 for CL, 0.75 for Q, 1.0 for V_C_, and 0.95 for V_P_^17^.

### 2.10 Projection of FIH dose and pharmacologically active dose

Based on previous results from non-clinical toxicology studies, cytokine release syndrome (CRS) was considered to be a potential adverse reaction to SIG001. Therefore, a strategy analogous to the minimum anticipated biological effect level (MABEL) approach was employed for the prediction of the first-in-human (FIH) dose (Muller et al., 2009). The threshold concentration of SIG001 that did not significantly increase cytokine release was determined from the *in vitro* cytokine release assay. The predicted PK parameters in humans were then used to simulate the PK profiles of SIG001 under different dosing regimens. The dosing regimen was selected such that the steady-state maximum concentration (C_max,ss_) of SIG001 approached but did not exceed this threshold. This dose was subsequently scaled down by a safety factor of 10 to derive the proposed FIH dose.

Meanwhile, the pharmacologically active dose (PAD) was predicted based on the PD results in tumor-bearing mice. The dose-response relationships of different SIG001 doses were analyzed to determine the efficacious dose range in mice. It is hypothesized that the same steady-state average serum concentrations (C_avg, ss_) were required to achieve equal anti-tumor efficacy in humans and mice (Eigenmann et al., 2016). The C_avg,ss_ in mice under the PAD model could be simulated based on the developed PK model. Subsequently, the predicted human PK model was used to simulate different dosing regimens, and human doses achieving comparable C_avg,ss_ values to the efficacious mouse exposure range were identified as the predicted PAD range. C_avg,ss_ was calculated using Equation 2:

$$C_{avg,ss}=\frac{{AUC}_{ss}}{\tau },$$ (2)

where AUC_ss_ is the steady-state area under the curve and τ is the dosing interval.

## 3 Results

### 3.1 SIG001 PK profiles in cynomolgus monkeys and mice

**FIGURE 1:**
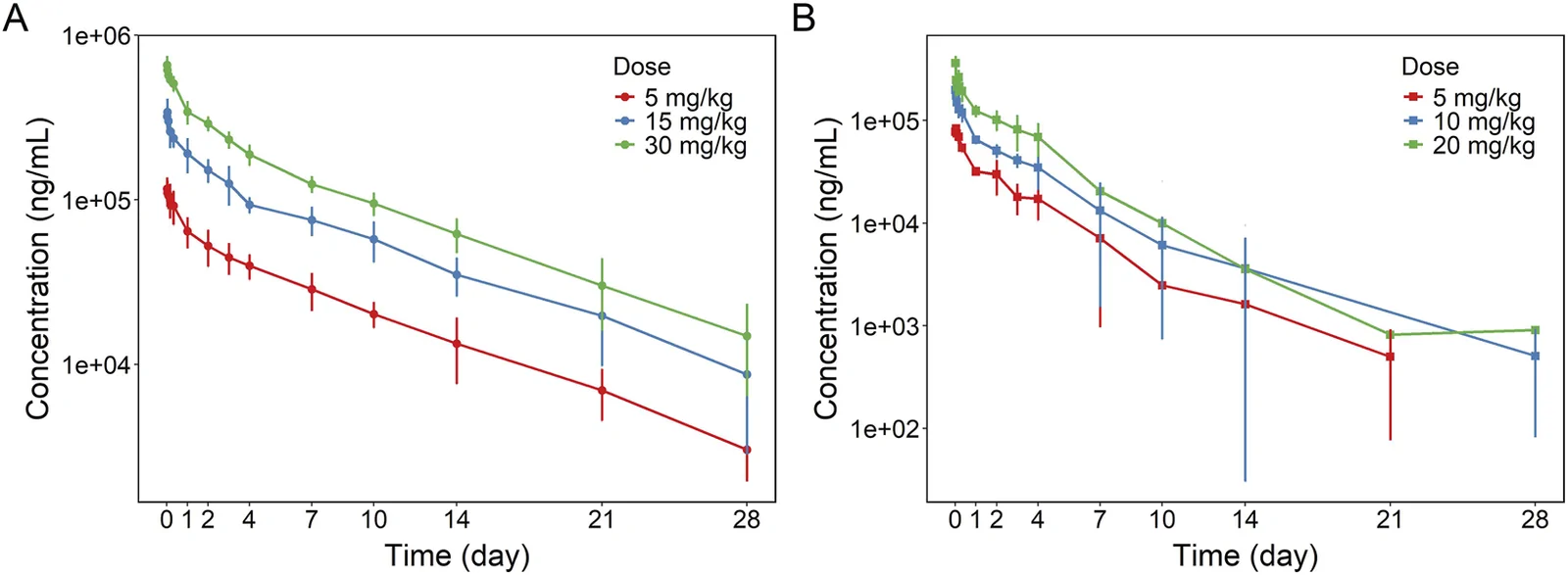
Time–concentration curves of SIG001 in **(A)** cynomolgus monkey and **(B)** mice after a single i. v. administration. Data are shown as the mean ± SD. SD, standard error.

A total of 251 observations and 1 BQL observation of SIG001 concentration were collected in the PK study of cynomolgus monkeys, and 216 observations and 54 BQL observations were collected in the PK study of mice. No obvious non-linear characteristics or target-mediated drug disposition was observed in the time–concentration curves, as shown in Figure 1, suggesting linear PK properties over the dose range of the PK studies. The half-life in was 153 h cynomolgus monkeys and 64.7 h in mice. No significant difference was found between the PK profiles of the male and female animals in both species.

### 3.2 Population PK modeling of SIG001 in cynomolgus monkeys and mice

**FIGURE 2:**
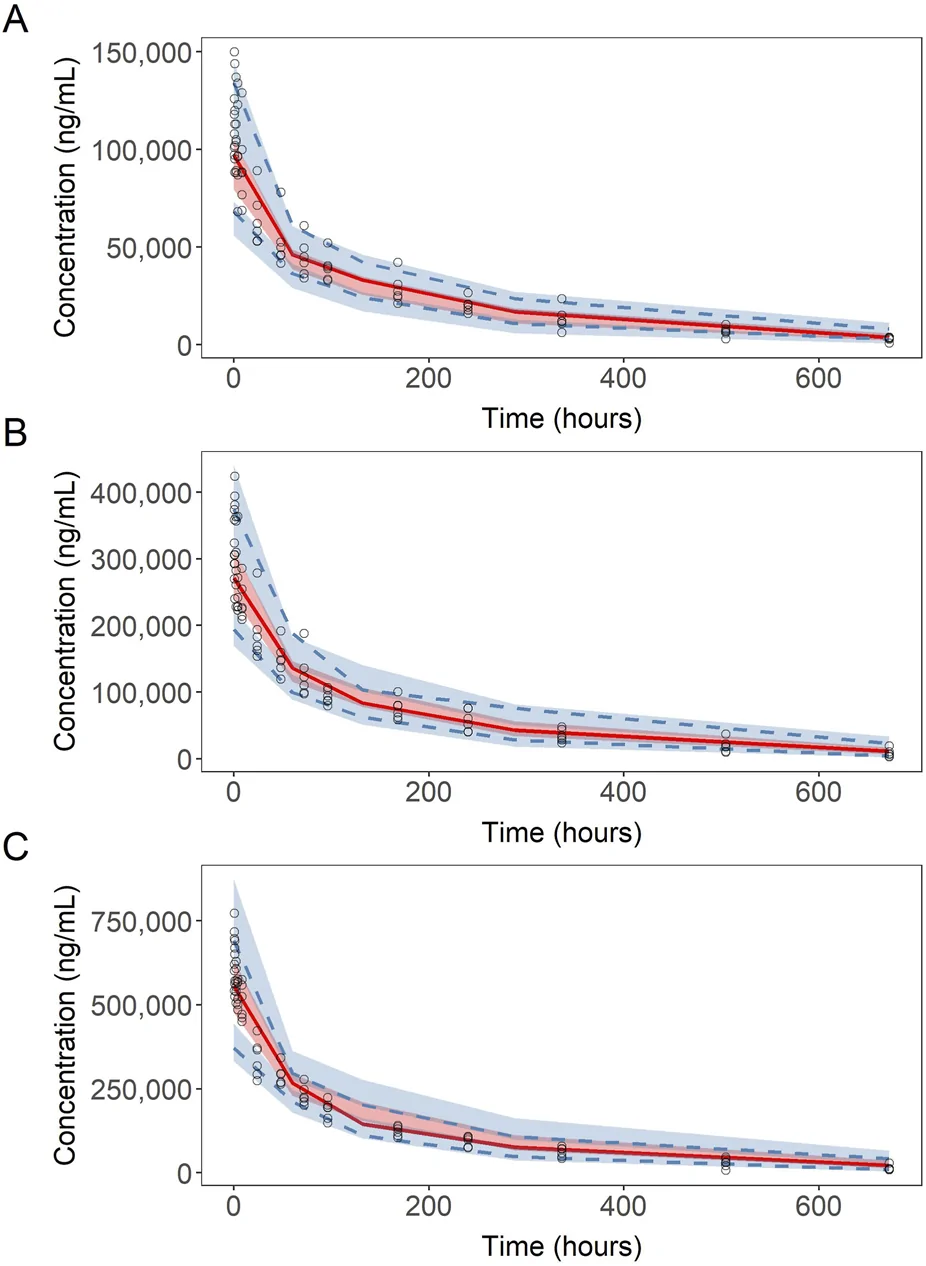
VPC for the PK model of SIG001 in cynomolgus monkeys after a single i. v. administration at **(A)** 5 mg/kg, **(B)** 15 mg/kg, and **(C)** 30 mg/kg. The red solid line represents the median line of the observations, and the red shaded area represents its 95% prediction interval. The red dashed lines represent the 5% and 95% percentiles of the observations, respectively, and the blue shaded areas represent their 95% prediction intervals, respectively. The circles represent observations. VPC, visual predictive check.

The PK behavior of SIG001 in cynomolgus monkeys was characterized using a two-compartment model incorporating proportional residual error. Table 1 presents the parameter estimates alongside bootstrap validation results. All RSE values were below 30%, and the bootstrap outcomes further corroborated the precision of the parameter estimates. Goodness-of-fit (GOF) plots (Supplementary Figure S1) of the Supplementary Material indicated that both population and individual predictions were symmetrically distributed around the line of identity, while conditional weighted residuals (CWRESs) predominantly fell within the range of −4 to 4. The predictive performance was further evaluated through a VPC based on 1,000 simulations (Figure 2), which demonstrated that the model reliably described the observed data.

**TABLE 1:** Parameter estimates for the PK model of SIG001 in cynomolgus monkeys.

Parameter	Estimate (RSE)	Bootstrap results	
		Median (RSE)	90% CI
CL (mL/h/kg)	0.386 (4.7%)	0.385 (4.6%)	0.357–0.414
V_C_ (mL/kg)	47.2 (3.3%)	47.1 (3.3%)	44.6–49.7
Q (mL/h/kg)	1.05 (17.0%)	1.07 (18.4%)	0.80–1.42
V_P_ (mL/kg)	31.4 (7.9%)	31.7 (8.0%)	27.6–36.1
IIV CL (%)	18.8 (14.7%)	18.3 (18.1%)	13.4–22.6
IIV V_C_ (%)	16.2 (19.1%)	15.8 (15.6%)	9.2–20.4
Proportional error (%)	12.6 (5.3%)	12.4 (12.4%)	11.2–13.5

CI, confidential interval; IIV, inter-individual variability; RSE, relative standard error.

**FIGURE 3:**
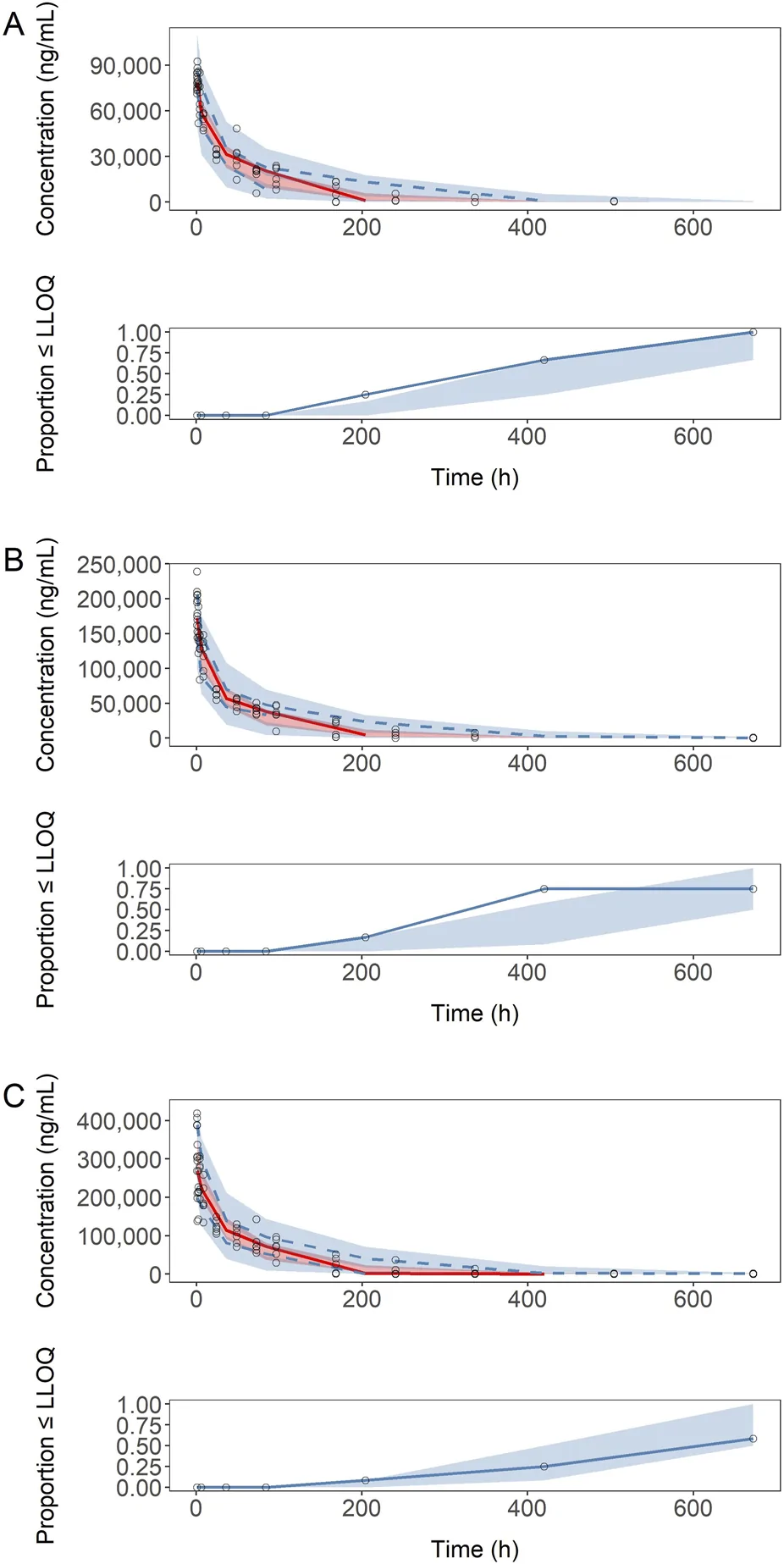
VPC for the PK model of SIG001 in mice after a single i. v. administration at **(A)** 5 mg/kg, **(B)** 10 mg/kg, and **(C)** 20 mg/kg. Upper panel: the red solid line represents the median line of the observations, and the red shaded area represents its 95% prediction interval. The red dashed lines represent the 5% and 95% percentile of the observations, respectively, and the blue shaded areas represent their 95% prediction intervals, respectively. The circles represent observations. Lower panel: the blue area represents 95% confidence intervals of the fraction of BQL observations, and the solid blue line and circles are the observed fraction of BQL samples. VPC, visual predictive check; BQL, below the quantification limit.

Similarly, the PK profile of SIG001 in mice was optimally characterized by a two-compartment model exhibiting proportional residual variability. Parameter estimates and bootstrap results are summarized in Table 2, with RSE values and bootstrap confidence intervals reflecting satisfactory estimation precision. GOF plots (Supplementary Figure S2) illustrated adequate concordance between predictions and observations, with all CWRESs remaining within the range of −4 to 4. Notably, at later time points (approaching 28 days), CWRESs exhibited a slight increase, likely attributable to a higher proportion of below-the-quantification-limit (BQL) data. VPC results from 1,000 simulations (Figure 3) affirmed acceptable predictive performance, with the majority of observed data points falling within the simulation-based prediction intervals and the proportions of BQL data being adequately captured by the model.

**TABLE 2:** Parameter estimates for the PK model of SIG001 in mice.

Parameter	Estimate (RSE)	Bootstrap results	
		Median (RSE)	90% CI
CL (mL/h/kg)	1.37 (3.7%)	1.36 (3.8%)	1.28–1.45
V_C_ (mL/kg)	57.7 (3.1%)	57.2 (3.4%)	54.0–60.4
Q (mL/h/kg)	3.48 (15.8%)	3.76 (22.5%)	2.84–5.34
V_P_ (mL/kg)	32.9 (5.8%)	33.0 (8.8%)	28.5–38.3
IIV CL (%)	43.4 (5.4%)	43.7 (14.5%)	39.7–47.8
Proportional error (%)	20.6 (6.0%)	20.0 (6.8%)	17.5–22.9

CI, confidential interval; IIV, inter-individual variability; RSE, relative standard error.

### 3.3 Tumor growth inhibition of SIG001 in tumor-bearing mice

**FIGURE 4:**
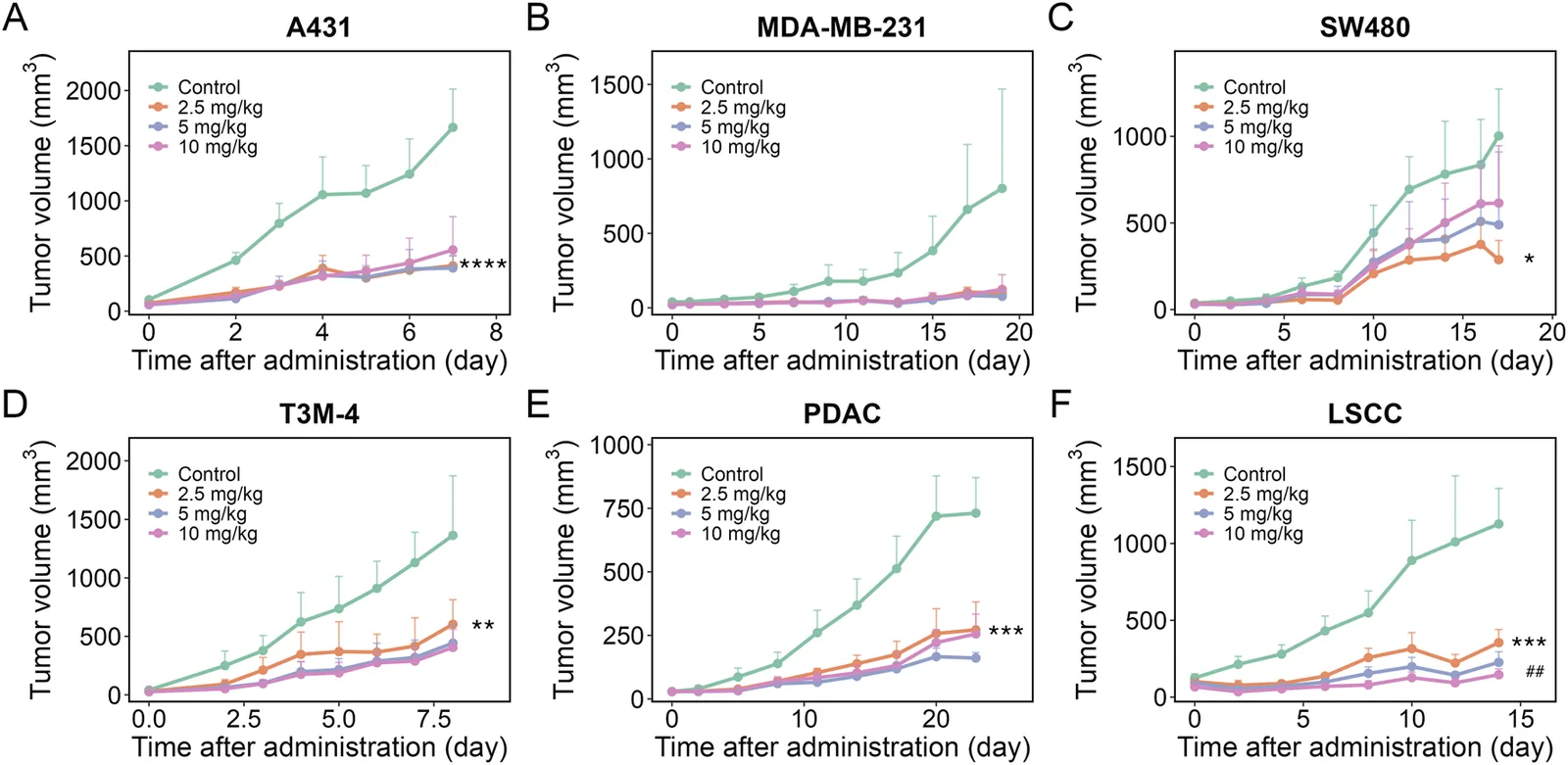
The tumor inhibition effect of SIG001 at 2.5, 5 and 10 mg/kg in **(A)** A431 **(B)** MDA-MB-231 **(C)** SW480 and **(D)** T3M-3 CDX as well as **(E)** PDAC and **(F)** LSCC PDX. CDX: cell-derived xenograft; PDX: patient-derived xenograft. *p < 0.05, **p < 0.01, ***p < 0.001, ****p < 0.0001 stand for control vs. 2.5 mg/kg group; ^**##**^ *p* < 0.01 stand for 2.5 mg/kg vs. 10 mg/kg group.

The tumor growth inhibition efficacy of SIG001 in various preclinical xenografts was evaluated, as shown in Figure 4. Significant effects of treatment, time, and treatment-by-time interaction were observed via REML analysis, indicating that SIG001 significantly altered tumor growth trajectories compared with the control groups. Although the 10 mg/kg group showed significantly greater tumor inhibition than the 2.5 mg/kg group in the LSCC model, this difference was not observed in other xenograft models. Overall, the tumor inhibition effects of 2.5, 5, and 10 mg/kg SIG001 were generally comparable, suggesting that near-maximal efficacy might be achieved within the dose range of 2.5–10 mg/kg.

### 3.4 Projection of FIH dose of SIG001

The cytokine release from human PBMCs induced by SIG001 was presented in Supplementary Figure S3 of Supplementary Material. Data showed that at a concentration of 50 μg/mL and within an incubation period of 96 h, SIG001 did not induce a significant increase in the levels of cytokines, including IL-2, IL-4, CXCL10, IL-1β, TNF-α, CCL2, IL-17A, IL-6, IL-10, IFN-γ, IL-12p70, IL-8, and TGF-β1, compared with the control and IVIG group. This indicated that SIG001 does not significantly stimulate cytokine elevation at concentrations up to 50 μg/mL, suggesting a relatively low risk of inducing CRS under this threshold.

**FIGURE 5:**
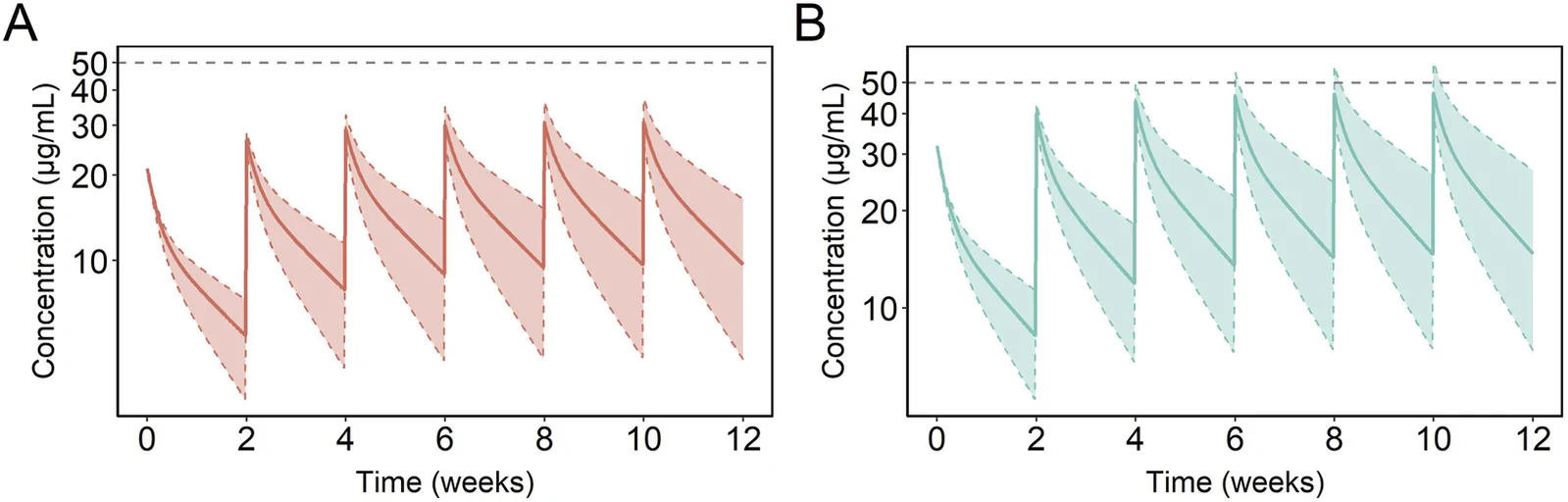
Simulated PK profiles of SIG001 in humans with dose regimens of **(A)** 1.0 mg/kg Q2W and **(B)** 1.5 mg/kg Q2W. The solid line is the median of predicted concentration, and the area is the 90% prediction interval. The grey dashed lines represent the threshold of 50 μg/mL.

The PK parameters in humans were derived based on the parameter estimates obtained in cynomolgus monkeys through allometric scaling, where CL was 0.194 mL h^-1^·kg^-1^, V_C_ was 47.2 mL kg^-1^, Q was 0.44 mL h^-1^·kg^-1^, and V_P_ was 26.4 mL kg^-1^, respectively. The predicted half-life of SIG001 in humans was approximately 11 days, and a dosing interval of 2 weeks was selected in the simulation, as shown in Figure 5. With a 30% IIV of CL and a 10% proportional residual variability, the predictions for the 1.0 mg/kg dose regimen were all below the 50 μg/mL threshold, which was considered a relatively safe concentration level based on the *in vitro* cytokine release assay. Furthermore, the median of predicted C_max,ss_ for the 1.5 mg/kg dose regimen was 45 μg/mL, and only the upper limit of the prediction interval exceeded 50 μg/mL. Taking a safety factor of 10-fold into consideration, a dose regimen of 0.15 mg/kg Q2W was selected as the FIH dose.

### 3.5 Projection of human pharmacologically active dose of SIG001

Since there was no clear dose-dependent tumor inhibition among the three dose groups (2.5, 5, and 10 mg/kg) across the xenograft models, linear or nonlinear extrapolation via PK/PD modeling to estimate the dose required for complete tumor inhibition could be biased. Instead, the HED corresponding to 2.5 mg/kg and 10 mg/kg in mice was calculated using murine PK parameters and the predicted human PK parameters, which could be considered a PAD range in humans. The C_avg,ss_ values in mice were 1.25 mg/mL at 2.5 mg/kg and 5.01 mg/mL at 10 mg/kg, i.e., under the dose regimen of the PD studies, and the corresponding AUC_ss_ were 420.0 mgh·mL^-1^ and 1,683.4 mgh·mL^-1^, respectively. Based on simulations with the predicted human PK parameters, the human PAD range was derived to be 1.8–7.0 mg/kg with a dosing interval of 2 weeks.

## 4 Discussion

Although targeted therapy and immunotherapy have greatly improved the treatment of cancers, the lack of effective novel targets means that immunotherapeutic approaches benefit only limited types of cancers and subsets of patients. SIA-IgG has been shown to be widely expressed and to play an important role in the progression of epithelial-derived malignancies (Tang et al., 2018; Huang et al., 2023; Wang et al., 2020; Fan et al., 2023), which account for over 80% of all cancer incidences (Brücher, 2025), and SIG001, which specifically targets SIA-IgG, holds the potential to become a broad-spectrum anti-tumor therapeutic antibody. In this study, the preclinical PK properties of SIG001 in cynomolgus monkeys and mice were investigated for the first time, and its anti-tumor efficacy was also evaluated in various xenografts. Moreover, pharmacometric modeling approaches were adopted to provide insights for the clinical translation to support the FIH trial design.

In the exploratory analysis of PK data, the PK behavior of SIG001 after a single i. v. dose exhibited notable linear elimination characteristics in both cynomolgus monkeys and mice, with no apparent nonlinear elimination or target-mediated drug disposition (TMDD) properties observed in the tested dose range. Moreover, as SIA-IgG is predominantly expressed in tumors and minimally distributed in non-tumor-bearing mice and cynomolgus monkeys, it was challenging to obtain relevant target information from the animals in the PK studies. With no observed TMDD characteristics during the terminal elimination phase of the PK curves and no target-binding kinetic parameters for SIG001 available, incorporating TMDD into the current model would lead to overparameterization. To avoid this issue, the TMDD model was finally not included, and a two-compartment model was better supported by the data and was also fit-for-purpose, though a TMDD model has been reported to be beneficial in translation studies (Haraya et al., 2017). Based on the modeling exploration, a two-compartment model with first-order elimination adequately described the PK profile of SIG001 in cynomolgus monkeys and mice and was thus selected as the final PopPK structural model. Taking robustness, parameter estimation accuracy, and overall fit of the model into consideration, IIV for CL and V_C_ was estimated in the cynomolgus monkey PK model. IIV for CL was 18.8% and V_C_ was 16.2%, indicating relatively low variability in the PK parameters of SIG001 in cynomolgus monkeys. However, in the PK model of mice, each individual provided at most two blood concentration observations, precluding reliable estimation of IIV for all parameters, and therefore, only IIV for CL was estimated.

The PK properties of SIG001 in humans were predicted solely from cynomolgus monkey PK parameters using allometric scaling. Although several animals have been used to evaluate the PK properties at the preclinical stage, cynomolgus monkeys are the most widely used species to predict human PK using an allometric scaling approach due to the similarity in FcRn-binding affinity against therapeutic mAbs (Yeung et al., 2009), and it is reported that single-species allometric scaling using cynomolgus monkeys showed even better prediction accuracy of human PK characteristics than multiple-species allometric scaling (Deng et al., 2011). Nevertheless, it is important to note that species differences, target biology, and possible nonlinear clearance in patients would still lead to uncertainty in the prediction of human PK. The higher SIA-IgG expression in tumor patients than in cynomolgus monkeys would probably lead to differences in the clearance of SIG001, especially higher target-mediated clearance, which is generally predominant at lower concentration levels.

Compared to the no observed adverse effect level (NOAEL), the MABEL approach is recognized as a safer way to select the FIH dose for therapeutic monoclonal antibody drugs (Muller et al., 2009; Yao et al., 2025). SIG001 demonstrated a favorable safety profile in nonclinical toxicology assessments, where the highest non-severely toxic dose (HNSTD) in cynomolgus monkeys was 200 mg/kg, while in mice, 200 mg/kg only resulted in a mild increase in liver weight without associated histopathological changes (data not published yet). Nevertheless, a small number of animals died within 24 h after the second, third, or fourth dose, which may have been related to immune responses. Therefore, a MABEL-guided, model-informed strategy was used to support the projection of the FIH starting dose. As part of this strategy, an *in vitro* PBMC cytokine release assay was performed as a supportive safety assessment. Considering the nonclinical safety profile, pharmacological activity, and predicted clinical exposure of SIG001, a concentration of 50 μg/mL was selected to evaluate potential cytokine release liability in human PBMCs. This concentration was determined to be pharmacologically relevant and conservative, derived from the C_avg_ of an intermediate efficacious regimen of 5 mg/kg in mice.

Depending on the mechanisms and associated pharmacological responses, the threshold level could typically be EC10, EC20, or EC50 at which the drug elicits a corresponding biological effect in predicting the FIH dose through the MABEL approach (Matsumoto et al., 2024). The main mechanism of action of SIG001 does not involve direct stimulation of immune cells to release cytokines, and its target SIA-IgG is predominantly expressed in tumors with low levels in healthy human cells. Thus, SIG001 is theoretically not supposed to activate cytokine release from immune cells and only a single concentration level of 50 μg/mL was tested in human PBMCs. The results showed that SIG001 at 50 μg/mL did not significantly stimulate the release of any cytokines, falling below the theoretical MABEL. Consequently, concentration levels below this 50 μg/mL threshold are expected to offer a satisfactory safety profile.

As a first-in-class therapeutic antibody, there is a lack of evidence on the PK variability of SIG001 in humans. Based on the variability observed in the PK parameters of other monoclonal antibody drugs, IIV for CL was set at 30% and the proportional residual error at 10% in the simulations, which represents a moderate level of variability (Dirks and Meibohm, 2010). In addition, for monoclonal antibody drugs that exert their pharmacological effects by specifically neutralizing a target, the MABEL can also be estimated based on receptor occupancy (RO) (Matsumoto et al., 2024). Since SIG001 primarily acts by binding to SIA-IgG on the surface of tumor cell membranes, and quantifying antigen levels on solid tumor membranes presents significant challenges and inaccuracies, the RO-related data are not yet available in this study, and eventually the dose projection was not made through the RO approach, which was considered a limitation of this study. Nevertheless, since the primary consideration in the FIH trial is ensuring the safety of participants, the current FIH dose projection can still provide evidence of clinical safety and is therefore fit-for-purpose.

As for the anti-tumor efficacy of SIG001, although it exhibited considerable tumor growth inhibition across dose groups in most tumor-bearing mouse xenograft models, no significant dose-dependent effect was observed at the experimental doses of 2.5, 5, and 10 mg/kg, which was another limitation of the study. On one hand, the results may suggest that the pharmacological effect of SIG001 may have reached saturation, although it did not completely inhibit tumor growth at doses above 2.5 mg/kg. On the other hand, substantial inter-individual variability in tumor growth and drug response, along with experimental variability, may have contributed to relatively high variation in certain experimental groups (such as the SW480 xenograft model), which could obscure the assessment of the dose–response relationship. Given the lack of significant enhancement in anti-tumor efficacy between 2.5 and 10 mg/kg and since PK/PD models could not be developed, further extrapolation to higher doses, whether with linear or nonlinear methods, would introduce considerable uncertainty. Therefore, a dose range of 2.5–10 mg/kg was finally employed to predict the human PAD based on reasonable assumptions (Eigenmann et al., 2016).

## 5 Conclusion

In summary, the PK properties of SIG001 in cynomolgus monkeys and mice were investigated, PopPK models were developed, and human PK parameters were further predicted via allometric scaling. A dose regimen of 0.15 mg/kg was projected as the FIH dose to avoid the risk of inducing CRS, and the human PAD was predicted to be in the range of 1.8–7.0 mg/kg. The model-based approach provided important reference points for designing the FIH trial of the first-in-class therapeutic antibody SIG001. As the clinical trial progresses, additional clinical data and learn-and-confirm iterative cycles will continue to inform subsequent research and development.

## Data Availability

The raw data supporting the conclusions of this article will be made available by the authors, without undue reservation.
