# Supplementary material for: Preclinical pharmacokinetic modeling and clinical translation of SIG001, a novel anti-cancer therapeutic antibody targeting on cancer-derived sialylated IgG
Source: Front Pharmacol. 2026 Sep 1;17:1904283. doi: 10.3389/fphar.2026.1904283 (PMC13574649; doi:10.3389/fphar.2026.1904283)
Supplement: Supplementary file 1 [file Supplementaryfile1.docx]

**Table S1.** PD study designs of SIG001 in tumor-bearing mice

| Xenograft | Implantation | Group | Dosing | Tumor volume measurement |
| --- | --- | --- | --- | --- |
| Human epidermoid carcinoma A-431 CDX | 2×10^6^ cells per animal | Control group (PBS, n=8)  SIG001 2.5 mg/kg (n=8)  SIG001 5 mg/kg (n=8)  SIG001 10 mg/kg (n=8) | i.v. administration at 0, 3, 5 d | 0, 2, 3, 4, 5, 6, 7 d |
| human triple-negative breast cancer MDA-MB-231 CDX | 2×10^6^ cells per animal | Control group (PBS, n=7)  SIG001 2.5 mg/kg (n=7)  SIG001 5 mg/kg (n=7)  SIG001 10 mg/kg (n=7) | i.v. administration at 0, 4, 8, 12, 16 d | 0, 1, 3, 5, 7, 9, 11, 13, 15, 17, 19 d |
| human pancreatic cancer T3M-4 CDX | 2×10^6^ cells per animal | Control group (PBS, n=9)  SIG001 2.5 mg/kg (n=10)  SIG001 5 mg/kg (n=10)  SIG001 10 mg/kg (n=10) | i.v. administration at 0, 3, 6 d | 0, 2, 3, 4, 5, 6, 7, 8 d |
| human colorectal cancer line SW480 CDX | 4.5×10^6^ cells per animal | Control group (PBS, n=6)  SIG001 2.5 mg/kg (n=6)  SIG001 5 mg/kg (n=6)  SIG001 10 mg/kg (n=6) | i.v. administration at 0, 5, 9, 12, 15 d | 0, 2, 4, 6, 8, 10, 12, 14, 16, 17 d |
| lung squamous cell carcinoma PDX | 2×2×2 mm^3^ tumor tissue per animal | Control group (PBS, n=6)  SIG001 2.5 mg/kg (n=6)  SIG001 5 mg/kg (n=6)  SIG001 10 mg/kg (n=6) | i.v. administration at 0, 4, 8, 12 d | 0, 2, 4, 6, 8, 10, 12, 14 d |
| pancreatic ductal adenocarcinoma PDX | 2×2×2 mm^3^ tumor tissue per animal | Control group (PBS, n=6)  SIG001 2.5 mg/kg (n=6)  SIG001 5 mg/kg (n=6)  SIG001 10 mg/kg (n=6) | i.v. administration at 0, 4, 8, 12, 16, 20 d | 0, 2, 5, 8, 11, 14, 17, 20, 23 d |

**Figure S1.** GOF plots for the PK model of SIG001 in cynomolgus monkeys, showing (A) observations versus individual predictions, (B) observations versus population predictions, (C) CWRES versus population predictions and (D) CWRES versus time after treatment. GOF goodness-of-fit, CWRES conditional weighted residual error.

**Figure S2.** GOF plots for the PK model of SIG001 in mice, showing (A) observations versus individual predictions, (B) observations versus population predictions, (C) CWRES versus population predictions and (D) CWRES versus time after treatment. GOF goodness-of-fit, CWRES conditional weighted residual error.

**Figure S3.** Cell cytokine release from human peripheral blood mononuclear cells after incubated with PBS, IVIG, RP215 and SIG001. (A) IL-4, (B) IL-2, (C) CXCL10, (D) IL-1β, (E) TNF-α, (F) CCL2, (G) IL-17A, (H) IL-6, (I) IL-10, (J) IFN-γ, (K) IL-12p70, (L) IL-8, (M) TGF-β1. ns stands for not significant.
